# Inference of gene regulatory networks based on directed graph convolutional networks

**DOI:** 10.1093/bib/bbae309

**Published:** 2024-06-27

**Authors:** Pi-Jing Wei, Ziqiang Guo, Zhen Gao, Zheng Ding, Rui-Fen Cao, Yansen Su, Chun-Hou Zheng

**Affiliations:** Key Laboratory of Intelligent Computing & Signal Processing of Ministry of Education, Institutes of Physical Science and Information Technology, Anhui University, 111 Jiulong Road, 230601, Anhui, China; Key Laboratory of Intelligent Computing & Signal Processing of Ministry of Education, School of Computer Science and Technology, Anhui University, 111 Jiulong Road, 230601, Anhui, China; Key Laboratory of Intelligent Computing & Signal Processing of Ministry of Education, School of Computer Science and Technology, Anhui University, 111 Jiulong Road, 230601, Anhui, China; Key Laboratory of Intelligent Computing & Signal Processing of Ministry of Education, Institutes of Physical Science and Information Technology, Anhui University, 111 Jiulong Road, 230601, Anhui, China; Key Laboratory of Intelligent Computing & Signal Processing of Ministry of Education, School of Computer Science and Technology, Anhui University, 111 Jiulong Road, 230601, Anhui, China; Key Laboratory of Intelligent Computing & Signal Processing of Ministry of Education, School of Artificial Intelligence, Anhui University, 111 Jiulong Road, 230601, Anhui, China; Key Laboratory of Intelligent Computing & Signal Processing of Ministry of Education, School of Artificial Intelligence, Anhui University, 111 Jiulong Road, 230601, Anhui, China

**Keywords:** directed graph convolutional network, gene regulatory network, local augmentation strategy, sequence features, dynamic update strategy

## Abstract

Inferring gene regulatory network (GRN) is one of the important challenges in systems biology, and many outstanding computational methods have been proposed; however there remains some challenges especially in real datasets. In this study, we propose Directed Graph Convolutional neural network-based method for GRN inference (DGCGRN). To better understand and process the directed graph structure data of GRN, a directed graph convolutional neural network is conducted which retains the structural information of the directed graph while also making full use of neighbor node features. The local augmentation strategy is adopted in graph neural network to solve the problem of poor prediction accuracy caused by a large number of low-degree nodes in GRN. In addition, for real data such as *E.coli*, sequence features are obtained by extracting hidden features using Bi-GRU and calculating the statistical physicochemical characteristics of gene sequence. At the training stage, a dynamic update strategy is used to convert the obtained edge prediction scores into edge weights to guide the subsequent training process of the model. The results on synthetic benchmark datasets and real datasets show that the prediction performance of DGCGRN is significantly better than existing models. Furthermore, the case studies on bladder uroepithelial carcinoma and lung cancer cells also illustrate the performance of the proposed model.

## Introduction

Gene regulatory network (GRN) is an important mechanism for maintaining life processes, controlling biochemical reactions and regulating compound levels, and plays an important role in organism and system. Inferring GRN will help us to understand the molecular mechanism of organisms more deeply, and then reveal the essential rules of a large number of biological processes in organisms [1]. GRN can be used in multiple tasks; for example, it can be used to derive novel biological hypotheses for molecular interactions, guide the experimental design of new experiments and identify biomarkers for complex diseases [2].

Although a substantial number of known biological regulatory relationships are documented in various databases, they still fall significantly short in representing the multitude of interactions and complex relationships present in real biological systems. Depending solely on manual experimentation to validate potential regulatory relationships one by one is undoubtedly unrealistic. Various researchers have proposed many computational methods for inferring GRN. The existing GRN inference methods mainly include correlation methods [3], Boolean network methods [4], knowledge-based methods [5], Bayesian network methods [6], differential equation methods [7], neural network methods, etc. [8, 9].

In recent years, with the development of deep learning and neural networks, more and more methods based on neural networks have emerged. CNNC [10], based on convolutional neural networks (CNNs), uses single-cell expression data to infer the relationship between genes. TDL [11] proposed two experimental models, one is based on the LSTM of the Recurrent Neural Network (RNN) to extract temporal feature information, and the other is based on 3D-CNN to extract spatial feature information. DGRNS [12] uses both CNN and RNN to infer accurate GRNs from single-cell transcriptomic data via deep learning models. However, it is seldom using deep learning method to infer the GRNs on bulk dataset, and the CNN and RNN used in the above methods do not perform well in processing non-Euclidean data such as GRN [13].

GRGNN [14] introduces graph neural networks (GNNs) into the study of GRN for the first time. It converts the GRN inference into a graph classification task. However, the GNN used by GRGNN does not consider the directionality of gene regulation, and stipulates that a certain gene can only be TF or target gene. But in reality, there are many gene nodes in the actual GRN that are both TF and target gene. As a result, this method can only infer the presence of regulatory relationships between genes but cannot accurately determine the direction of regulation. Additionally, it may result in the loss of some regulatory relationships.

In this work, we propose a novel GRN inference method DGCGRN based on directed graph convolutional network (DGCN). The DGCN is introduced to overcome the problem that traditional GCN cannot handle directed graph data well. And for reducing the impact of low-degree nodes, a local augmentation strategy is adopted to generate the additional enhanced features, in which the generative model Conditional Variational Autoencoder (CVAE) is used to learn the feature distribution of neighbored nodes on the condition of central node’s feature distribution [15, 16]. Furthermore, for real dataset *E.coli*, two types of sequence features are extracted by two ways: one is the hidden features extracted by Bi-GRU model, and another is the calculated statistical characteristics. Then the sequence features are integrated with gene expression features and enhanced features to further prediction. In the process of neural network prediction, a dynamic update strategy is adopted to convert the possibility of a regulatory relationship among nodes obtained in each cycle into the weight of an edge, and a new weighted graph is used to replace the original graph to guide the follow-up training process, which can improve the predictive performance of the model.

## Method

**Figure 1:**
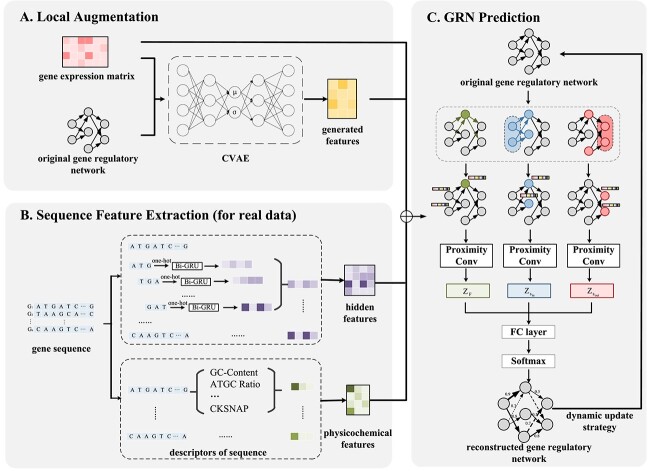
The framework of DGCGRN model. A: Using conditional variational auto-encoders to implement local augmentation of GNNs. B: Extracting gene sequence features and biological sequence features. C: Using DGCNs to predict GRNs.

The proposed DGCGRN method is a novel GRN inference method based on DGCN [17]. The scheme of DGCGRN is shown in Figure 1 and it contains three modules: (A) local augmentation module which is used to generate the enhanced features, (B) sequence feature extraction module which is used to extract sequence features for real organism data and (C) GRN prediction module which is used to inference GRN based on directed convolutional network.

### Local augmentation strategy

The traditional method of local augmentation of machine learning data is to augment part of the entire graph structure while ignoring the local augmentation of individual node information. However, for the characteristics of GRN data with many low-degree nodes, the existing local augmentation methods are not applicable. Therefore, we use augment operations within a certain range of the graph structure to generate neighborhood features through a generative model conditioned on local structure and node features [18].

**Figure 2:**
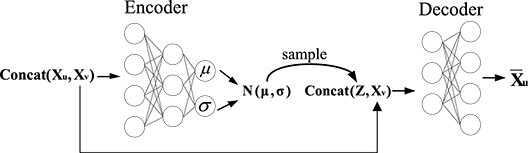
CVAE structure diagram.

The generative model CVAE is built based on multilayer perceptron (MLP) (Figure 2), and the encoder and decoder consist of two layers of MLP. Here, we define the central node as $v$ and its feature distribution as $X_{v}$; the neighbors and their feature distribution are $u$ ($u \in N_{v}$) and $X_{u}$, respectively. The node pair features $(X_{u},X_{v})$ are as input in the encoding stage. In the decoding stage, the latent variable $z$ extracted from $N(0,I)$, and the feature vector $X_{v}$ of the central node $v$ are as the input of the CVAE decoder. Finally, the generated feature vector $\bar{X}_{v}$ is obtained.

### Sequence feature extraction

In real organism *E.coli* data, the dimension of gene expression features is low. As we know, in machine-learning-based models, features are key to determine the effectiveness of models. Low-dimensional features may cause the graph CNN unable to be fully expressed. In this study, considering the importance of sequence, the sequence features are extracted, which can not only solve the problem of low-dimensional data and learn the contextual relationship, but also simulate the interaction between various parts of the gene sequence [19].

Two different methods were used for extracting sequence features. The first method involves utilizing the Bidirectional GRU (Bi-GRU) model to extract hidden features of sequences. Bi-GRU is a model based on RNN, which preserves previous information through gating mechanism when processing input data, enabling it to process time series data. Compared with other RNN models, Bi-GRU avoids the problems of gradient vanishing and gradient exploding. Its simpler structure and fewer parameters contribute to heighten computational efficiency and reduce model complexity. In order to extract more comprehensive features and achieve superior robustness and predictive performance, we opt for Bi-GRU. Given the intricate local contextual relationships inherent in gene sequences, we segment the complete gene sequences into multiple k-mer fragments [20]. Then each unique k-mer fragment is embedded and represented by one-hot encoding [21]. Finally, the sequence characteristics of the gene are learned through Bi-GRU.

Another way involves calculating the feature descriptors or representation of physicochemical properties, termed as physicochemical features (Figure 1B). There are different gene regulatory relationships that vary across different organisms, which lead to diverse traits exhibited by organisms. The variability stems from factors such as the content of specific types of bases in genes, or the different bending and folding angles of the entire peptide chain. Consequently, we further infer the gene regulatory relationships in various organisms by calculating physicochemical features. The descriptors of physicochemical features are calculated as follows:

(1) Z-curve [22]: For any given DNA sequence, a unique Z-curve corresponding to it can be found. The coordinates of each node are expressed as follows: 


(1) \begin{align*}& \begin{aligned} &X_{n}=\left(A_{n}+G_{n}\right)-\left(C_{n}+T_{n}\right)=2\left(A_{n}+G_{n}\right)-n\\ &Y_{n}=\left(A_{n}+C_{n}\right)-\left(T_{n}+G_{n}\right)=2\left(A_{n}+C_{n}\right)-n\\ &Z_{n}=\left(A_{n}+T_{n}\right)-\left(C_{n}+G_{n}\right)=2\left(A_{n}+T_{n}\right)-n \end{aligned},\end{align*}

where $X_{n},Y_{n},Z_{n}\in [-n,n],n=1,2,...,n$;

(2) GC Content [23]: the proportion of Guanine (G) and Cytosine (C) in the gene:


(2) \begin{align*}& \begin{aligned} \text{ GC Content} =\frac{\sum G+\sum C}{\sum A+\sum C+\sum G+\sum T} \end{aligned}\end{align*}

(3) ATGC Ratio [24]: the ratio between the bases (A+T) and (C+G) in the DNA double-strand:


(3) \begin{align*}& \begin{aligned} \text{ AT/GC Ratio }=\frac{\sum A+\sum T}{\sum G+\sum C} \end{aligned}\end{align*}

(4) NAC (Nucleic Acid Composition) [25, 26]: frequency of each nucleic acid type in a nucleotide sequence: 


(4) \begin{align*}& f(t)=\frac{N(t)}{N}, t \in\{A, C, G, T\},\end{align*}

where ${N(t)}$ is the number of nucleotide types $t$, $N$ is the length of the nucleotide sequence;

(5) CKSNAP (Composition of k-spaced Nucleic Acid Pairs) [27]: The feature encoding calculates the frequency of splitting nucleotide pairs of k residues. For each k-space, there are 16 types of nucleic acid pair composition (‘A...A’, ‘A...C’, ‘A...G’, ‘A...T’, ‘C...A’, ‘C...C’, ‘C...G’, ‘C...T’, ‘G...A’, ‘G...C’, ‘G...G’, ‘G...T’, ‘T...A’, ‘T...C’, ‘T...G’, ‘T...T’). In this study, we select k = 0, so the definition of CKSNAP is as follows: 


(5) \begin{align*}& \left(\frac{N_{AA}}{N_{\text{total }}}, \frac{N_{AC}}{N_{\text{total }}}, \frac{N_{AG}}{N_{\text{total }}}, \ldots, \frac{N_{AT}}{N_{\text{total }}}\right)_{16},\end{align*}

where $N_{AA}$ means the times of $AA$ appearance and $N_{\text{total}}$ represents the total number of 0-spaced nucleic acid pairs in sequence.

Finally, the extracted features from the two methods are concatenated as input features for the neural network.

### Directed graph convolutional network

The traditional GCN is often used to process undirected graph structure data [28]. If the directed graph structure data such as GRN is processed, it will be relaxed to undirected graph, which will undoubtedly lose a lot of information. So we introduce a DGCN to directly process directed graph data. In order to better understand the DGCN, some related concept definitions of directed graph CNNs are introduced as follows.

#### Concept definition

Definition 1. Directed graph Define $G=(V,E)$ as a directed graph, where $V={v_{1},...,v_{n}}$ is the set of vertices, $E={e_{1},e_{2},\ldots ,e_{m}}$ is the set of edges and each directed edge $e\in E$ is composed of source node $v_{i}$ and target node $v_{j}$, representing the directed path from $v_{i}$ to node $v_{j}$.

**Figure 3:**
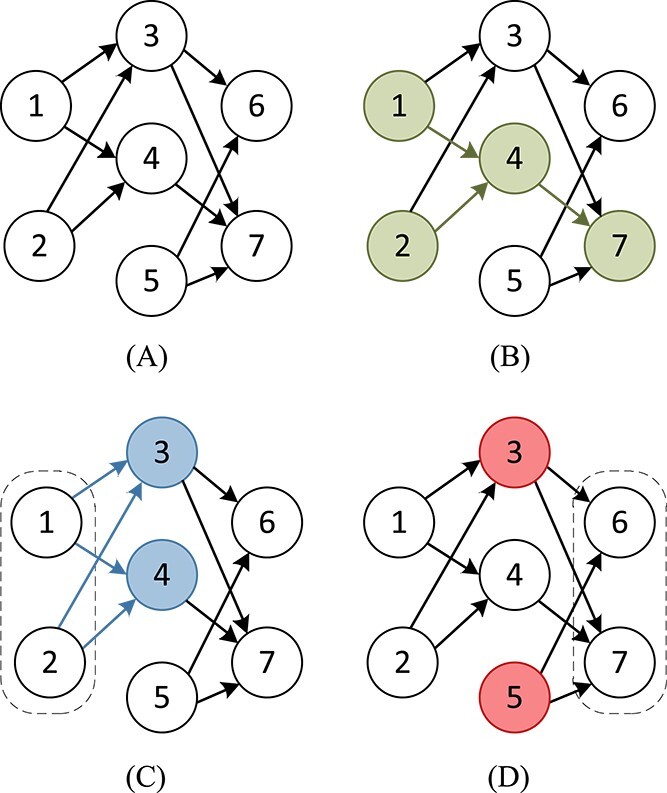
First- and second-order proximity.

Definition 2. First- and second-order edges in directed graph Given a directed graph $G=(V,E)$, for an ordered pair $v_{i},v_{j}\in V$, if $(v_{i},v_{j})\in E$, we define the edge between this ordered pair $(v_{i},v_{j})$ as a first-order edge. For example, in Figure 3(B), there are first-order edges between nodes $(v_{1},v_{4})$, $(v_{2},v_{4})$and $(v_{4},v_{7})$. If there is a node $k\in V$ that satisfies the ordered pair $(v_{k},v_{i})$ and $(v_{k},v_{j})\in E$ or $(v_{i},v_{k})$ and $(v_{j},v_{k})\in E$, we define the edge between the ordered pair $(v_{i},v_{j})$ as a second-order edge. For instance, as shown in Figure 3(C), there is an edge between the node pair $(v_{1},v_{3})$, $(v_{1},v_{4})$, we define there is a second-order edge between the node pair $(v_{3},v_{4})$. In a similar way, $(v_{3},v_{5})$ in Figure 3(D) are also defined as second-order edge. Specially, the second-order edge may not be known.

Definition 3. First-order proximity matrix In order to model the first-order proximity, we define the first-order approximation $A_{F}$ in the following form:

(6) \begin{align*}& A_{F}(i, j)=A^{s y m}(i, j),\end{align*}

where $A^{sym}$ is the symmetric matrix of edge weights matrix $A$. If it is not defined, the weights of all edges are set to 1, that is, $A$ is the adjacency matrix of the graph structure. $A_{F}(i,j)=0$, if there is no edge from node $v_{i}$ to $v_{j}$ (or from node $v_{j}$ to $v_{i}$).

Definition 4. Second-order proximity matrix In a directed graph $G$, for nodes $v_{i}$ and $v_{j}$, a second-order in-degree approximation $A_{S_{in}}(i,j)$ and an out-degree approximation $A_{S_{out}}(i,j)$ are defined:

(7) \begin{align*} A_{S_{in}}(i,j)=\sum_{k} \frac{A_{k,i} A_{k,j}}{\sum_{v} A_{k,v}} \end{align*}

(8) \begin{align*} A_{S_{out}}(i,j)=\sum_{k} \frac{A_{i,k} A_{j,k}}{\sum_{v} A_{v,k}}, \end{align*}

where $A$ is the weighted adjacency matrix of graph $G$ and node $k,v\in V$. $A_{S_{in}}(i,j)$ is the normalized summation of the edge weights of the arrays of nodes $v_{i}$ and $v_{j}$. The array is defined as nodes which target to $v_{i}$ and $v_{j}$, i.e. $\sum _{k} A\{i \leftarrow k \rightarrow j\}$. The larger the value of $A_{S_{in}}(i,j)$, the second-order of nodes $v_{i}$ and $v_{j}$ the higher the degree of similarity. Similarly, $A_{S_{out}}(i,j)$ calculates the second-order out-degree similarity between nodes $v_{i}$ and $v_{j}$, i.e. $\sum _{k} A\{i \rightarrow k \leftarrow j\}$. If there is no common node from or to nodes $v_{i}$ and $v_{j}$, then the second-order similarity between these two nodes is set to 0.

#### Directed graph convolution

After obtaining the three approximate matrices $A_{F}$, $A_{S_{in}}$ and $A_{S_{out}}$, the spectral convolution of the first- and second-order approximate matrices is 


(9) \begin{align*}& \begin{gathered} Z_{F}=f_{F}\left(X, \tilde{A}_{F}\right)=\widetilde{D}_{F}^{-\frac{1}{2}} \tilde{A}_{F} \widetilde{D}_{F}^{-\frac{1}{2}} X \Theta\\ Z_{S_{\text{in }}}=f_{S_{\text{in }}}\left(X, \tilde{A}_{S_{\text{in }}}\right)=\widetilde{D}_{S_{\text{in }}}^{-\frac{1}{2}} \tilde{A}_{S_{\text{in }}} \widetilde{D}_{S_{\text{in }}}^{-\frac{1}{2}} X \Theta\\ Z_{S_{\text{out }}}=f_{S_{\text{out }}}\left(X, \tilde{A}_{S_{\text{out }}}\right)=\widetilde{D}_{S_{\text{out }}}^{-\frac{1}{2}} \tilde{A}_{S_{\text{out }}} \widetilde{D}_{S_{\text{out }}}^{-\frac{1}{2}} X \Theta \end{gathered},\end{align*}

where $\tilde{A}$ is an adjacency matrix with self-loops($\tilde{A}=A+I$), $\widetilde{D}_{F}=\operatorname{diag}\left (\sum _{j}^{n} \tilde{A}_{F}(i,j)\right )$, $\widetilde{D}_{S_{in}}=\operatorname{diag}\left (\sum _{j}^{n} \tilde{A}_{S_{in}}(i,j)\right )$, $\widetilde{D}_{S_{out}}=\operatorname{diag}\left(\sum_j^n \tilde{A}_{S_{out}}(i,j)\right)$, $X$ is feature matrix, $\Theta $ is a shared trainable weight matrix.

The $Z_{F}$, $Z_{S_{in}}$ and $Z_{S_{out}}$ obtained by the convolution formula not only obtains a large amount of first-order and second-order neighborhood feature information, but also retains the structural information of the directed graph.

### Dynamic update strategy

In the graph convolutional network, the weight information of topological graph edges is usually calculated through the message-passing mechanism. When the input graph structure is weightless, no weight information will be transmitted during the training process of the model. In the message-passing mechanism, the messages received by each node are sent by its adjacent nodes. When the information aggregation is performed on the weighted graph structure, the nodes can select the adjacent nodes that need to be aggregated more according to the weight information of the edge’s information, so it is possible to more accurately infer the relationship between the node pair.

Dynamic update strategy [29] is adopted to convert the score of regulatory relationship between nodes obtained in each iteration during the model training process into the probability (0-1) of the existence of regulatory relationship, and the probability value is used as the weight of the directed edge between the node pair. Therefore, the original input unweighted graph can be converted into a weighted graph, and the weighted graph can be used to replace the original input unweighted graph.

## Experimental results

### Benchmark datasets

To test the ability of the proposed DGCGRN method on GRN inference, we use simulated benchmark datasets and real organism expression data. The simulated datasets are from DREAM (Dialogue for Reverse Engineering Assessments and Methods) challenges [30], which provide benchmark datasets to validate the ability of GRN reconstructed methods. It includes DREAM4 [31–33] and DREAM5 [34] challenges. In this study, five simulated datasets from DREAM4 and two datasets from DREAM5 are collected, the details of which are summarized in Table 1. Two datasets from DREAM5 are one simulated dataset (in silico) and one real expression dataset (*E.coli*). Furthermore, the real datasets are *E.coli* expression data in different condition containing cold stress, heat stress and oxidative stress datasets, the details of which are summarized in Table 2. And the 4747 *E.coli* gene sequence data are collected from the RegulonDB database [35]. We can find a total of 4747 *E.coli* gene sequence data from the RegulonDB database [35].

**Table 1:** DREAM4 & DREAM5 dataset

Dataset	Network	Gene	Expression data	Known regulatory interaction
DREAM4	Network1	100	210	176
	Network2			249
	Network3			195
	Network4			211
	Network5			193
DREAM5	Net1-in silico	1463	805	4012
	*E.coli*	4511		2066

**Table 2:** *E.coli* data

Network	Gene	Sample	Time Point	Known regulatory interaction
Cold stress	1484	3	8	3080
Heat stress				
Oxidative stress			11	

### Performance metrics

In order to evaluate the performance of our model, we use 10 times of 5-fold cross-validation and calculate the Area Under the ROC curve (AUROC) as our final performance metrics, where AUROC is the area under the ROC curve drawn with the false positive rate (FPR) as the abscissa and the true positive rate (TPR) as the ordinate. 


(10) \begin{gather*} \mathrm{TPR}=\frac{\mathrm{TP}}{\mathrm{TP}+\mathrm{FN}} \end{gather*}

(11) \begin{gather*} \mathrm{FPR}=\frac{\mathrm{FP}}{\mathrm{FP}+\mathrm{TN}} \end{gather*}

(12) \begin{gather*} \mathrm{ACC}=\frac{\mathrm{TP}+\mathrm{TN}}{\mathrm{TP}+\mathrm{FP}+\mathrm{TN}+\mathrm{FN}}, \end{gather*}

where TP refers to the number of links correctly identified, TN refers to the number of false links correctly identified, FP refers to the number of links incorrectly identified and FN refers to the number of false links incorrectly identified.

### Selection of sequence feature extraction methods

**Figure 4:**
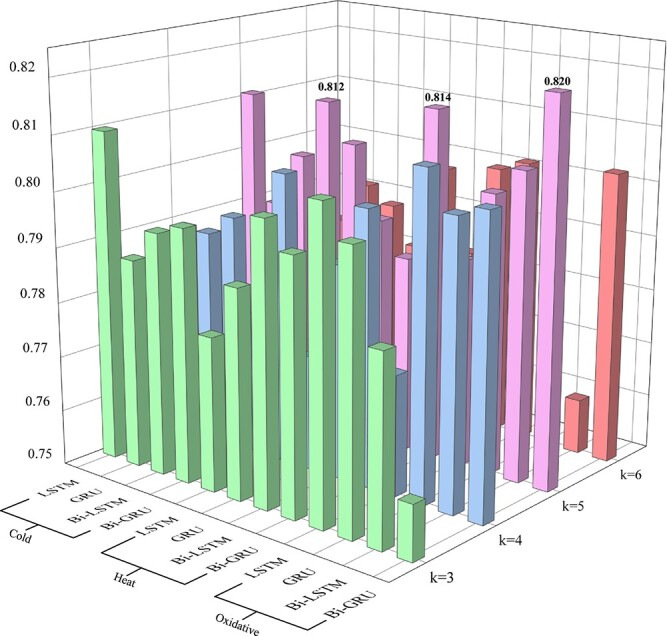
The results of different length k-mer fragments and different RNN models in each network.

In this study, for considering the importance of gene sequence and enhancing features in real data, we extract sequence features of *E.coli* by machine learning methods. RNN-based models are suitable for processing sequence data; in order to select a better method to extract sequence features, we test and compare several commonly used RNN-based schemes: LSTM, GRU, Bi-LSTM and Bi-GRU. The k-mer segments with one-hot encoding are the input data of these schemes. Then grid search method is used to select the optimal scheme and best $k$ of k-mer (Figure 4).

By comparing the effects of k-mer fragments of different lengths in different RNN models, it can be observed that the best results on each *E.coli* dataset are achieved when the gene is divided into 5-mer fragments and feature extraction of the gene sequence is performed using Bi-GRU.

### Parametric analysis

Since the model on *E.coli* data includes both feature enhancement and sequence feature extraction modules, we conduct parameter analysis using *E.coli* data as an example. The dimensions of the generated features obtained by the local enhancement strategy and the sequence features extracted by Bi-GRU will affect the performance of the model. We conduct some experiments to determine the most appropriate dimensions. Firstly, in the local augmentation strategy, the feature dimension of neighboring nodes learned by the generative model is determined by the number of nodes in the last layer of the decoder. We determine the optimal feature dimensions by experimenting different numbers of nodes. Since the expression feature dimension in *E.coli* data is 24(or 33), during analysis, the generated feature dimension is initially set to 20, which is close to the number of expression features, and then increases by 10 dimensions.

When using Bi-GRU to extract sequence features from *E.coli*, it is necessary to perform zero-padding on sequences with shorter lengths during embedded representation, since genes have varying sequence lengths. Therefore, if the sequence feature dimension obtained by Bi-GRU is too high, more invalid data will be obtained from genes with shorter sequences.

**Figure 5:**
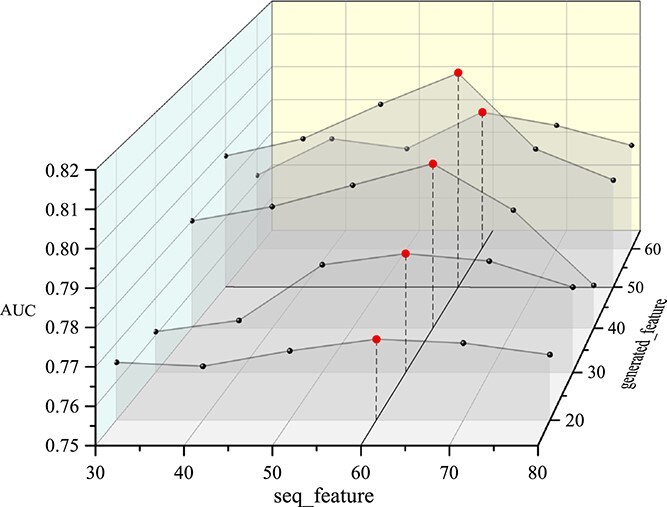
Generative features and sequence feature results of different dimensions in cold network.

As can be seen from Figure 5, when the dimension of the sequence features extracted by the neural network is too high, the prediction results decline. Through experiments, it was finally determined that the node generation feature dimension is 50, and the number of sequence features extracted by Bi-GRU is 60.

### Ablation study

In this section, we use several sets of ablation experiments to verify the effectiveness of each module of the model in inferring GRN. Here, we take the *E.coli* dataset as an example and conduct six experiments on the E.coli dataset (Table 3):

**Table 3:** The results of *E.coli* ablation experiments, by comparing the AUC values of each group of experiments, prove the effectiveness of each module

	Method	Cold	Heat	Oxidative
E.1	GCN	0.644	0.633	0.643
E.2	DGCN	0.718	0.732	0.740
E.3	+CVAE	0.774	0.775	0.777
E.4	+CVAE	0.791	0.795	0.801
	+Seq feature			
E.5	+CVAE	0.795	0.810	0.790
	+dynamic update strategy			
E.6	+CVAE			
	+Seq feature	0.812	0.814	0.820
	+dynamic update strategy			

We conducted six experiments on the *E.coli* dataset (Table 3):

Experiments 1 (E.1): It uses undirected GCN to predict the directed graph data structure.

E.2: It uses directed graph convolution network.

E.3: It adds a graph network local augmentation strategy based on DGCN.

E.4: It adds sequence and biological features based on E.3.

E.5: It uses the dynamic update strategy based on E.3.

E.6: It adds sequence and biological features based on E.5.

The results of E.2 show that the DGCN can better process directed graph structured data. The results of E.3 show that the local augmentation strategy can solve the problem of low-degree nodes well. And the results of E.4, E.5 and E.6 show the effect of sequence features and dynamic update strategy.

In order to verify the effectiveness of dynamic update strategy and it with different strategies, we conduct ablation experiments on other datasets (DREAM4, DREAM5 and BLCA), the results are shown in Supplementary Tables S1 and S2. The experimental results indicate that the dynamic update strategy is effective to various datasets.

### Computing time

In addition to predictive performance, for evaluating the computational complexity of neural network models, we calculated the running time of DGCGRN, GNIPLR [36] and MMFGRN [37] on the DREAM4, *E.coli* and DREAM5 datasets, respectively. The computing time is measured on a 16GB RAM, Intel(R) Core(TM) i7-10870H CPU computer, and the results are shown in Table 4. From the comparison of running time with other methods, it can be seen that DGCGRN is far superior in computational time compared with other methods.

**Table 4:** Comparing computing time with other methods

Method	DREAM4	*E.coli*	DREAM5
DGCGRN	7min31s	53min28s	6h37min
GNIPLR	15min34s	3h15min	8h47min
MMFGRN	20min17s	3h58min	9h7min

### Comparing with other methods

**Figure 6:**
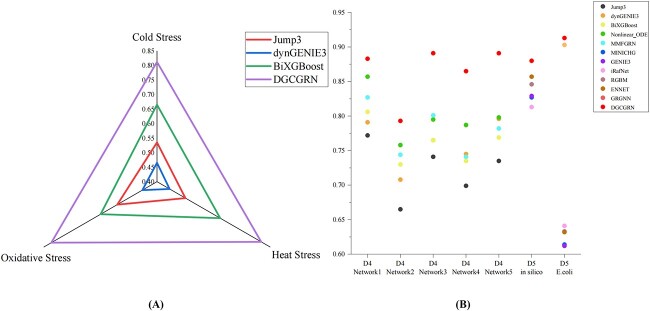
Comparison of AUC values with other method’s results. (A) The result in *E.coli* data. (B) The results in DREAM4 and DREAM5 data. D4 represents the DREAM4 dataset, and D5 represents the DREAM5 dataset.

In order to test the performance of proposed method DGCGRN, it is compared with some existing state-of-the-art methods in different datasets. Firsty, for *E.coli* data, since some methods do not experiment on *E.coli* data, we compared DGCGRN with Jump3 [38], dynGENIE3 [7] and BiXGBoost [39]. Five times 5-fold cross-validation experiments on the *E.coli* dataset are conducted and the average AUC of all methods are calculated. As shown in Figure 6(A), it is obvious that the prediction results of our method are better than other methods.

Furthermore, for simulation data of DREAM4 dataset and simulated datasets in DREAM5, due to the lack of sequence information, module B of DGCGRN is not used. The results are shown in Figure 6(B). The results show its better performance than other methods, indicating the advantage of DGCGRN in inferring GRN. The comparison results of AUPR with other methods can be found in Supplementary Table S3; it can be seen that the AUPR value of DGCGRN is significantly higher than other methods.

## Case study

Compared with data such as *E.coli*, GRNs in human species are more complex and exhibit different regulatory relationships in different tissues and under varying conditions. Most complex diseases are usually caused by dysregulation of certain functional modules and pathways, and rewiring genetic pathways may have an impact on disease research [40]. Cancer accounts for a higher proportion and has a higher mortality rate. Therefore, reconstruction cancer GRNs can help to explore the regulation mechanism of cancer, to determine potential biomarkers and drugs. In this study, we used DGCGRN to infer the GRN for two cancers bladder urothelial carcinoma (BLCA) and lung cancer (LUNG), and explored deeper analysis.

### The experiment and analysis of BLCA

Bladder cancer is one of the most common malignant tumors and is among one of the top 10 most common tumors. In our country, bladder cancer has the highest incidence rate among all genitourinary system tumors. The known network information of BLCA comes from the hsa05219 pathway in Kyoto Encyclopedia of Genes and Genomes (KEGG), which includes regulatory information for bladder cancer. The intersection genes between this pathway and BLCA data are selected from TCGA database, which result in a sub-network of 10 genes and 7 regulatory edges [36]. It should be noted that due to the inability to obtain gene sequence data for BLCA, we used sequence data from the human reference genome as the gene sequence for BLCA. As we know, there are genetic mutations in sequence data in cancer patients. To verify the impact of mutations on the inference of GRNs, we conducted a simulated mutation experiment on the reference genome with genetic site mutations frequency $10^{-3}$ (Supplementary Table S4). The experimental results show that the predictive performance of the model did not significantly change after simulating gene site mutations, so we use the human reference genome as sequence information in our model DGCGRN for human disease data.

**Figure 7:**
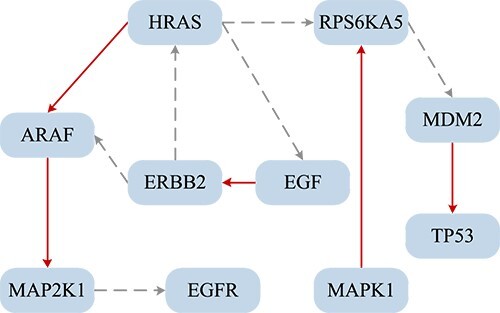
BLCA dataset network topology. The red line represents the predicted known regulatory relationship, while the gray dashed line represents false positives or newly discovered regulatory relationships.

The BLCA network constructed by the DGCGRN is shown in Figure 7, The red lines indicate the predicted known regulatory relationship, such as the established regulatory relationship in KEGG, and the gray lines indicate false positives or newly discovered regulatory relationships.

By comparing the AUC values of other methods (Table 5), we can see that the prediction results of our method are better than other methods.

**Table 5:** AUC values for GNPLR, GENIE3-RF-sqrt, GENIE3-EF-sqrt, NIMEFI, GENIMS, PLSNET and NARROMI methods

Method	AUC
GENIE-RF-sqrt	0.51
GENIE-ET-sqrt	0.52
NIMEFI	0.62
GENIMS	0.54
PLSNET	0.57
NARROMI	0.62
GNIPLR	0.66
DGCGRN	0.76

Since the known regulatory relationship is not always complete, the gray regulatory relationship in Figure 7 may be a false positive or a regulatory relationship that needs to be further studied. For the gray regulatory relationship, we further do literature study for verifying them.

For example, the regulatory relationship between *ERBB2* and *HARS* has not been verified in the hsa05219 pathway of KEGG, but our model predicts this regulatory connection. Seidlitz *et. al*. [41] has detected that the amplification of *ERBB2* significantly affects the expression of c-MYC-mediated target genes *CCND2*, *CDKN1A* and *THBS1* in the gastric cancer, which further leads to cancer mutations in genes such as HRAS (Gly13Asp). That is to say, in gastric cancer cells, *HRAS*, as the target gene, is indeed affected by the amplification and over expression of *ERBB2*, that is, there is such a regulatory relationship between the two genes.

Besides, Huang [42] has detected that wild-type *HRAS*, *NRAS* or *KRAS* have been shown to promote the proliferation of oncogenic RAS-driven cancer cell lines by mediating *EGF* signaling, which means that *HRAS* has a certain regulatory effect on *EGF*.

The new inferred gene regulatory relationship through model prediction can provide indications for subsequent research.

### The experiment and analysis of LUNG

**Figure 8:**
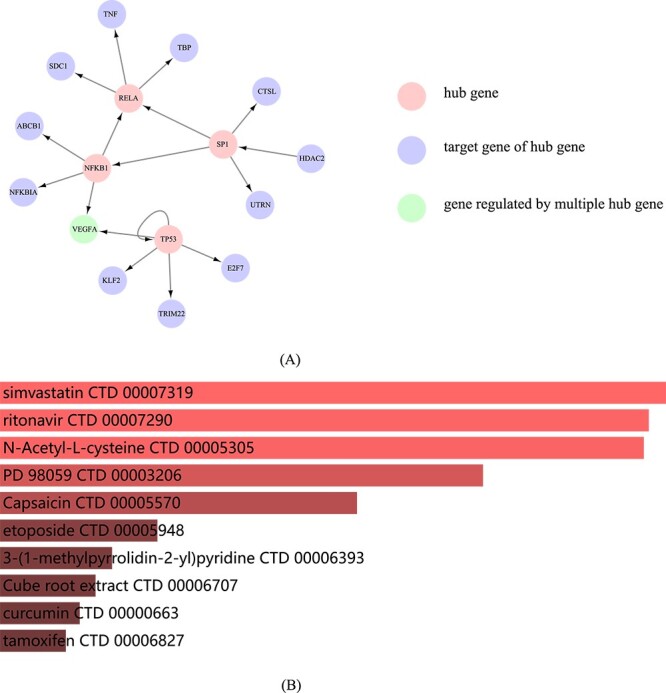
Results of lung cancer data case analysis. (A) The predicted hub genes and their regulatory relationships in the lung cancer data network. (B) Drug enrichment analysis of lung cancer.

To further validate the biological significance of DGCGRN, we reconstructed GRN using LUNG data [43] and employed in-depth analysis including prediction of biomarkers and enrichment analysis of potential therapeutic drugs. The lung cancer data contain 2478 genes and 8907 regulatory relationships. Compared with the BLCA dataset, the lung cancer data have a larger sample size and greater research significance. After reconstructing GRN, we selected four hub genes with the highest degree, and selected top five potential regulatory genes for each hub gene for visualization (Figure 8(A)).

The hub genes in Figure 8(A) have been fully validated through literature review. In human cancers, the somatic mutation of *TP53* is one of the most common mutations and is also potential markers [44]. The increased inflammation exhibited by *NFKB1* leads to the phenotype of cancer [45]. And *SP1* may regulate important related biomarkers such as *CD147* in lung cancer [46], while *RELA* promotes the proliferation of lung cancer cells [47]. In addition, for validating the importance of the predicted regulatory relationships, we manually validated the regulatory genes with high predicted scores for these four hub genes, and some of the regulatory relationships were validated in literature [48–50]. Taking *NFKB1* as an example, it has been reported that *NFKB1* can regulate *ABCB1* by encoding transcription factors [51]. It has been investigated that the common variants within *NFKB1* and *VEGFA* involved in sporadic breast cancer [52]. *NFKB1* and *RELA* are reported as hub genes in protein–protein network of downregulated genes in human lung squamous cell carcinoma [53]. The predicted potential regulatory relationship may need an in-deeper investigation in future.

Furthermore, we performed drug enrichment analysis on the hub genes with degree greater than 100 predicted by DGCGRN. Figure 8(B) shows the top 10 enriched potential drugs based on DsigDB obtained through hub genes. It has been confirmed that six out of the 10 drugs in Figure 8(B) can be used for the treatment of lung cancer. Simvastatin and Etoposide can be combined with other drugs to treat small cell lung cancer [54, 55]. Tamoxifen plays a certain role in anti-proliferation of non-small cell lung cancer [56], while Capsaicin is a new method for treating small cell lung cancer [57]. Ritonavir acts as a protease inhibitor to inhibit lung cancer cells by inhibiting survivin [58]. Curcumin and its derivatives play a versatile role in lung cancer therapy [59]. This indicates that these genes may serve as promising drug targets for further investigation and treatment of lung cancer which demonstrates the effectiveness of DGCGRN.

## Conclusion and discussion

In this study, we propose a model DGCGRN based on DGCNs, which are less used in the field of GRN reconstruction. For processing directed GRN, we adopt DGCN which is more suitable to infer directed GRN. Moreover, there are a large number low-degree nodes in the graph structure, which may restrict the expressive power of GNN; local feature augmentation is performed by CVAE to solve the problem. Furthermore, for real data, considering the importance of sequence in real organism, we capture the sequence features. To update the weight matrix according to the data in each iteration, a dynamic update strategy is conducted to improve the predictive performance of the model. For further verifying the performance of the proposed method on infering complex regulation relationship, we implement it to both simulated datasets DREAM4 and DREAM5 and real datasets *E.coli* data. The results show that the DGCGRN performs better than other baseline methods, which indicates that it can reconstruct GRN well. Case studies on real datasets bladder urothelial carcinoma and lung cancer also indicate that DGCGRN can infer some new regulatory relationships. And further analysis shows that it can help to infer hub genes accurately, and find therapeutic drugs for related diseases, which makes our work have broader application prospects.

However, there are still some areas that can be improved. The rapid development of gene sequencing technology has made the types of regulatory relationships between genes more diverse. It will be more useful if we can uncover the regulation type. And for data without known regulatory relationships, inferring regulatory networks directly from gene expression data can become more complex. Considering the importance of prior knowledge, we hope to combine methods based on prior knowledge to infer more specific types of regulatory relationships.

**Key Points**

- We develop DGCGRN based on directed graph convolutional neural networks, which can not only infer the regulatory relationships among genes, but also determine the directionality of the regulatory relationships.
- DGCGRN uses CVAE to perform local augmentation of the graph neural network to avoid the problem of low-degree nodes restricting the expressive power of the graph neural network.
- For real data, sequence features are combined which contains the features calculated by Bi-GRU and sequence descriptors features of genes.
- We use a dynamic update strategy in the neural network prediction process to update the weight matrix based on the results of each iteration of the neural network to improve the prediction performance.

## Supplementary Material

Supplementary_Data_bbae309

## Data Availability

The original data are available from the DREAM4 challenge (https://dreamchallenges.org/dream-4-in-silico-network-challenge/), DREAM5 challenge (https://dreamchallenges.org/dream-5-network-inference-challenge/), and RegulonDB (https://regulondb.ccg.unam.mx/). All codes and other corresponding data are freely available in GitHub repository https://github.com/thinpillow/dgcgrn.

## Author Biographies

**Pi-Jing Wei** is a lecturer in the Key Laboratory of Intelligent Computing & Signal Processing of Ministry of Education, Institutes of Physical Science and Information Technology, Anhui University, Anhui, China. Her main research interests include bioinformatics, synthetic biology, cancer data mining and machine learning.

**Ziqiang Guo** is currently a master degree candidate in the Key Laboratory of Intelligent Computing & Signal Processing of Ministry of Education, School of Computer Science and Technology, Anhui University, Anhui, China. His research interests include bioinformatics and machine learning.

**Zhen Gao** is a PhD candidate in the Key Laboratory of Intelligent Computing & Signal Processing of Ministry of Education, School of Computer Science and Technology, Anhui University, Anhui, China. Her current research interests are in mathematical and computational biology, and systems biology.

**Zheng Ding** is a master degree candidate in the Key Laboratory of Intelligent Computing & Signal Processing of Ministry of Education, Institutes of Physical Science and Information Technology, Anhui University, Anhui, China. His research interests include bioinformatics and machine learning.

**Rui-Fen Cao** is an associate professor in the Key Laboratory of Intelligent Computing & Signal Processing of Ministry of Education, School of Computer Science and Technology, Anhui University, Anhui, China. Her research interests include artificial intelligence, medical image processing and multimodal data fusion.

**Yansen Su** is a professor in the Key Laboratory of Intelligent Computing & Signal Processing of Ministry of Education, School of Artificial Intelligence, Anhui University, Anhui, China. Her specialized research areas are bioinformatics, systems biology, and artificial intelligence.

**Chun-Hou Zheng** is a professor at the Key Laboratory of Intelligent Computing & Signal Processing of Ministry of Education, School of Artificial Intelligence, Anhui University, Anhui, China. His research interests include pattern recognition, intelligent information processing and Bioinformatics.
