# Supplementary material for: Inference of gene regulatory networks based on directed graph convolutional networks
Source: Brief Bioinform. 2024 Jun 27;25(4):bbae309. doi: 10.1093/bib/bbae309 (PMC11209731; doi:10.1093/bib/bbae309)
Supplement: Supplementary_Data_bbae309 [file supplementary_data_bbae309.docx]

**Supplementary**

**Table S1.** Results of ablation experiments on dynamic update strategy in different datasets

| **Dataset** | **Network** | **With dynamic update strategy** | **Without dynamic update strategy** |
| --- | --- | --- | --- |
| *E.coli* | Cold | **0.812** | 0.791 |
|  | Heat | **0.814** | 0.795 |
|  | Oxidative | **0.820** | 0.801 |
| DREAM4 | Network1 | **0.889** | 0.854 |
|  | Network2 | **0.793** | 0.772 |
|  | Network3 | **0.891** | 0.863 |
|  | Network4 | **0.865** | 0.841 |
|  | Network5 | **0.891** | 0.852 |
| DREAM5 | Network1-in Silico | **0.880** | 0.847 |
|  | Network3-  *E.coli* | **0.913** | 0.881 |
| BLCA | -- | **0.755** | 0.721 |

**Note**: The experimental results are all AUC values of ten times of five-fold cross validation.

**Table S2**. Experiment on Changing Dynamic Update Strategies

| **Dataset** | **Network** | **E.1** | **E.2** | **E.3** | **E.4** | **Dynamic update strategy** |
| --- | --- | --- | --- | --- | --- | --- |
| *E.coli* | Cold | 0.783 | 0.780 | 0.791 | 0.796 | **0.812** |
|  | Heat | 0.792 | 0.791 | 0.796 | 0.799 | **0.814** |
|  | Oxidative | 0.794 | 0.789 | 0.797 | 0.803 | **0.820** |
| DREAM4 | Network1 | 0.863 | 0.857 | 0.865 | 0.870 | **0.889** |
|  | Network2 | 0.779 | 0.770 | 0.769 | 0.773 | **0.793** |
|  | Network3 | 0.876 | 0.864 | 0.869 | 0.879 | **0.891** |
|  | Network4 | 0.846 | 0.839 | 0.841 | 0.849 | **0.865** |
|  | Network5 | 0.876 | 0.861 | 0.871 | 0.880 | **0.891** |
| DREAM5 | Network1 - in Silico | 0.862 | 0.851 | 0.868 | 0.865 | **0.880** |
|  | Network3 - E. coli | 0.880 | 0.877 | 0.872 | 0.881 | **0.913** |

**Note:** The experimental results are all AUC values of ten times of five-fold cross validation.

**E.1**: Using **fixed weight** without dynamic update strategy. Generate a fixed weight matrix before the neural network begins training, and only use this fixed weight matrix during the subsequent training process of the model.

**E.2**: Using **random weight** without dynamic update strategy. The experimental method is the same as E.1.

**E.3**: Changing the order of dynamic update strategy by specific metric: During the training process of *n* epochs in a neural network, using a specific method to select the training epochs of the network for dynamic update strategy. For example, in *n* epochs, the first *n*/2 epochs use dynamic update strategy, while the latter *n*/2 epochs do not use dynamic update strategy, or dynamic update strategy are applied every other epoch. To achieve the goal of changing the order of dynamic update strategy.

**E.4**: Changing the order of dynamic update strategy randomly: In the training of a neural network for *n* epochs, randomly select several epochs to use dynamic update strategy. The number of epochs and which epochs are chosen for this strategy are determined randomly.

**Table S3.** Comparison of AUPR results with other methods

| **Method** | ***E.coli*** | | | **DREAM4** | | | | | **DREAM5** | |
| --- | --- | --- | --- | --- | --- | --- | --- | --- | --- | --- |
|  | Cold | Heat | Oxidative | Network1 | Network2 | Network3 | Network4 | Network5 | Network1  in Silico | Network3  *E.coli* |
| Jump3 | 0.014 | 0.013 | 0.021 | 0.270 | 0.110 | 0.200 | 0.180 | 0.174 | -- | -- |
| GENIE3 | -- | -- | -- | 0.338 | 0.309 | 0.277 | 0.267 | 0.114 | 0.291 | 0.093 |
| GENIE3-lag | 0.011 | 0.012 | 0.011 | 0.228 | 0.096 | 0.230 | 0.157 | 0.168 | -- | -- |
| BiXGBoost | 0.021 | 0.020 | 0.018 | 0.235 | 0.152 | 0.261 | 0.204 | 0.214 | -- | -- |
| Nonlinear_ODE | -- | -- | -- | 0.370 | 0.243 | 0.322 | 0.321 | 0.294 | -- | -- |
| iRafNet | -- | -- | -- | 0.552 | 0.337 | 0.414 | 0.421 | 0.298 | -- | -- |
| RGENIE | -- | -- | -- | -- | -- | -- | -- | -- | 0.343 | 0.104 |
| MMFGRN | -- | -- | -- | 0.393 | 0.251 | 0.367 | 0.331 | 0.332 | -- | -- |
| DGCGRN | **0.872** | **0.877** | **0.879** | **0.988** | **0.927** | **0.989** | **0.967** | **0.974** | **0.823** | **0.941** |

**Note**: The experimental results are all AUPR values.

**Table S4.** The experimental results of simulating gene mutations

| **Dataset** | **Network** | **Normal data** | **Gene mutation data** |
| --- | --- | --- | --- |
| *E.coli* | Cold | 0.812 | 0.807 |
|  | Heat | 0.814 | 0.810 |
|  | Oxidative | 0.820 | 0.813 |
| BLCA | -- | 0.755 | 0.749 |
| Lung Cancer | -- | 0.725 | 0.721 |

**Note**: The experimental results are all AUC values of ten times of five-fold cross validation.

**Normal data**: Refers to the results using sequence data of the human reference genome.

**Gene mutation data**: Refers to the results by simulating cancer gene mutations, which are randomly changing the nucleobase in genes, and the proportion of mutated gene sites is 0.1%.
